# Body composition and risk factors associated with sarcopenia in post-COVID patients after moderate or severe COVID-19 infections

**DOI:** 10.1186/s12890-022-02014-x

**Published:** 2022-06-08

**Authors:** Dulce González-Islas, Carlos Sánchez-Moreno, Arturo Orea-Tejeda, Samantha Hernández-López, Fernanda Salgado-Fernández, Candace Keirns-Davis, Susana Galicia-Amor, Esperanza Trejo-Mellado, Laura Gochicoa-Rangel, Armando Castorena-Maldonado

**Affiliations:** 1 Heart Failure and Respiratory Distress Clinic, Instituto Nacional de Enfermedades Respiratorias “Ismael Cosío Villegas”, Calzada de Tlalpan 4502 Col Sec XVI CP 14080 Del Tlalpan, Mexico City, Mexico; 2 Pulmonary Rehabilitation Department, Instituto Nacional de Enfermedades Respiratorias “Ismael Cosío Villegas”, Mexico City, Mexico; 3 Department of Pulmonary Physiology, Instituto Nacional de Enfermedades Respiratorias “Ismael Cosío Villegas”, Mexico City, Mexico; 4 Otorhinolaryngology Department, Instituto Nacional de Enfermedades Respiratorias “Ismael Cosío Villegas”, Mexico City, Mexico

**Keywords:** Post-COVID-19, Sarcopenia, Body composition, Invasive mechanical ventilation

## Abstract

**Background:**

Post-COVID-19 syndrome is characterized by diverse symptoms and abnormalities that persist beyond 12 weeks from the onset of acute COVID-19. Severity disease has been associated with more musculoskeletal alterations such as muscle weakness, dyspnea, and distance walking. The aim was to evaluate the impact of invasive mechanical ventilation (IMV) on body composition and investigate risk factors associated with sarcopenia in post-COVID-19 patients three months after moderate or severe COVID-19 infections.

**Methods:**

Cross-sectional study. 530 patients with PCR-confirmed diagnoses of moderate to severe COVID-19, > 18 years old, oxygen saturation ≤ 93%, PaO_2_/FiO_2_ ratio < 300, who required hospitalization and were discharged were included. We excluded those who died before the follow-up visit, declined to participate, or could not be contacted.

**Results:**

The mean age was 53.79 ± 12.90 years. IMV subjects had lower phase angle and handgrip strength and higher impedance index, frequency of low muscle mass, and low muscle strength than those without IMV. The risk factors of sarcopenia were > 60 years of age, diabetes, obesity, IMV, and prolonged hospital stay. The multivariate model showed that age > 60 years (OR: 4.91, 95% CI: 2.26–10.63), obesity (OR: 3.73, 95% CI: 1.21–11.54), and interaction between prolonged length of hospital stay and IMV (OR: 2.92; 95% CI: 1.21–7.02) were related to a higher risk of sarcopenia.

**Conclusion:**

Obesity and the interaction between prolonged length of hospital stay and IMV are associated with a higher risk of sarcopenia at 3 months after severe or moderate COVID-19 infection.

## Introduction

Coronavirus infection disease 2019 (COVID-19) is a viral infection caused by coronavirus-2 (SARS-CoV-2) leading to a severe acute respiratory syndrome in some people that induces a severe inflammatory state, oxidative stress, endothelial damage and microvascular injury, a pro-coagulant state, and maladaptation of the angiotensin-converting enzyme 2 pathway, all of which may contribute to post-COVID syndrome [1–3].

Post-COVID-19 syndrome has been characterized as symptoms and abnormalities persisting beyond 12 weeks from the onset of acute COVID-19 and not attributable to alternative diagnoses [4]. In addition, subjects with moderate or severe COVID-19 infections has more presence or persistence of symptoms such as fatigue or muscle weakness, myalgia, and reduction in quality of health and pulmonary function at six months after the acute infection [5, 6]. At 90 or more days after onset/hospitalization, around 38.4% of this population has been reported to have fatigue or muscle weakness, 33% dyspnea, and 9.7% myalgia [5].

In affected patients, the consequences can be observed at pulmonary, hematologic, cardiovascular, endocrine, renal, gastrointestinal and musculoskeletal levels, as well as and 49.7% developed malnutrition during hospitalization [8].

Regarding musculoskeletal consequences, Pironi et al. observed that subjects admitted to the intensive care unit had more frequency of weight loss > 5% (66.7% vs. 36.5%), as well, the prevalence of nutritional risk (95.7% vs. 67.3) than subjects admitted to intermediate care [7]. Similar results were found in other studies [8, 9]. Several of the factors that may contribute to more significant musculoskeletal alterations include the severity of disease, prolonged hospital stay, pro-inflammatory states and IMV. Huang et al. showed that at six months after acute COVID-19 infection, patients with moderate or severe COVID-19 infections who had undergone IMV compared to those who received oxygen therapy through high-flow nasal cannula had higher frequencies of muscle wasting (81% and 59%, respectively) and less distance walked in a 6 min walk test (29% and 22%, respectively) [6].

On other hand, low muscular strength or low muscle mass, as well as sarcopenia, are associated with an increased likelihood of adverse outcomes, including falls, fractures, physical disability, diminished capacity for the activities of daily living, and mortality [10–16].

Sarcopenia is a progressive and generalized disorder of skeletal muscle, and the diagnosis is confirmed by low muscular strength and low muscle mass [14].

Different factors contribute to sarcopenia and skeletal muscle disorder such as age, and pre-existing metabolic and inflammatory conditions like diabetes, pulmonary disease, and cardiovascular diseases, pro-inflammatory states, malnutrition, use of corticosteroids, prolonged hospital stay, and IMV [6, 17–24].

Different studies have shown skeletal muscle disorders [7–9] and the possible origins of them in acute COVID-19 [6, 17–24]. However, in post-COVID-19 patients, evidence about body composition alterations and risk factors for sarcopenia is still insufficient. The aim of the present study was to evaluate the impact of IMV on body composition and investigate the risk factors associated with sarcopenia in post-COVID-19 patients at three months after moderate or severe COVID-19 infections.

## Material and methods

A cross-sectional study was carried out at Instituto Nacional de Enfermedades Respiratorias “Ismael Cosío Villegas”, a level three teaching hospital in Mexico City. Data were obtained from ambulatory patient evaluations three months after acute COVID-19 infection during routine clinical care of post-Covid-19 subjects between June 1, 2020, and June 30, 2021.

Moderate to severe COVID-19 patients with PCR-confirmed diagnosis, > 18 years old, blood oxygen saturation ≤ 93% on room air, PaO_2_/FiO_2_ ratio of arterial partial pressure of oxygen to fraction of inspired oxygen < 300 who required hospitalization were discharged and, signed informed consent were included. We excluded those who died before the follow-up visit, declined to participate, or could not be contacted.

### Outcome measures

Body composition, anthropometry, pulmonary function, and clinical and demographic variables were performed by a qualified nutritionist and physician and standardized as part of routine studies in the post-COVID-19 clinical management provided to the patients who came to our Institute.

#### Anthropometry

Weight and height were measured according to the manual reference of anthropometric standardization [25]. All subjects wore light clothing and were barefoot. Body mass index was calculated by dividing the total body weight (kilograms) by the height squared (meters).

#### Body composition

Body composition was measured with whole-body bioelectrical impedance using four-pole multi-frequency equipment BodyStat QuadScan 4000 (BodyStat, Isle of Man, UK). The standard technique [26] was used: The measurements were all performed by the same operator, in the morning, in a comfortable area, free of drafts, and with portable electric heaters. The subjects were fasting and should not have exercised eight hours before or consumed alcohol 12 h before the study. During the entire study, the subject was in a supine position with arms separated from the trunk at about 30º and legs separated at about 45º.

The area was cleaned with alcohol, and electrodes were placed on the hand and ipsilateral foot. Appendicular skeletal muscle mass (ASMM) was assessed according to Sergi’s formula [27]: ASMM (Kg/m^2^) = [− 3.964 + (0.227*(Height^2^ (cm)/Resistance) + (0.095*Weight) + (1.384*Sex) + (0.064*Reactance) /Height (m^2^)].

#### Handgrip strength

Handgrip strength was measured using a mechanical Smedley Hand Dynamometer (Stoelting, Wood Dale, UK) according to the technique described in Rodriguez et al. [28].

#### Sarcopenia

Sarcopenia was defined according to EWGSOP2 as the presence of low muscle mass (in men ASMM < 20 kg and in women as ASMM < 15 kg) and low muscle strength (in men handgrip strength < 27 kg and in women handgrip strength < 16 kg) [14].

#### Respiratory muscle strength

Maximum inspiratory pressures (MIP) and maximum expiratory pressures (MEP) were measured based on ATS/ERS 2002 recommendations using MicroRPM equipment (CareFusion, Micromedical, UK) [29].

### Statistical analysis

Analyses were performed using the commercially available package STATA version 14 (Stata Corp., College Station, TX, U.S.A.). Categorical variables were expressed as frequencies and percentages. The Shapiro–Wilk test was used to assess the normality of continuous variables; normal continuous variables were expressed as mean and standard deviation, while non-normal variables were expressed as median and percentiles 25–75. A comparison among study groups (subjects with IMV and without IMV) was analyzed with X^2^ for categorical variables and unpaired Student's *t*-test or Mann Whitney U tests for continuous variables. A simple logistic regression model to evaluate the risk factors associated with sarcopenia was performed to estimate the odds ratio (OR) and 95% Confidence Intervals (CI). Risk factors with *p*-value < 0.20 in the unadjusted model were included in the full multivariable regression model, and then stepwise selection (*p*-value ≤ 0.20 as “in” criteria and *p*-value ≥ 0.05 as “out” criteria) was applied to generate the final model. These associations were adjusted for sex and, diabetes, VIH, COPD and, PaO_2_/FiO_2_ ratio. A *p* < 0.05 was considered to be statistically significant.

## Results

A total of 530 post-COVID subjects were included. The age median age was 53.79 ± 12.90 years, and 60.94% were males.

Subjects managed with IMV had a higher prevalence of ischemic cardiopathy, a longer hospital stay (25 [17–36] vs. 9 [7–13], *p* < 0.001), lower arterial oxygen saturation (81.5 [69.1–90] vs. 86.7 [81–91], *p* < 0.001), PO_2_ (49.7 [36.1–65.3] vs. 55 [45.8–67.5], *p* < 0.010), and PaO_2_/FiO_2_ ratio (129.52 [90.93–176.09] vs. 226.42 [172.99–264.76], *p* < 0.001), besides more frequency of acute respiratory distress syndrome moderate-severe (86% vs. 41.33%, *p* < 0.001) at admission than subjects who were not treated with IMV. Duration of IMV were 17 [10–29] days.

Regarding body composition, subjects exposed to IMV had lower phase angle, handgrip strength, and higher impedance index, frequency of low muscle mass, and low muscle strength (Table 1).

**Table 1:** Clinical characteristics and body composition of Post-COVID-19 patients subdivided by invasive mechanical ventilation utilization

	All n = 530	Invasive mechanical ventilation n = 329	No Invasive mechanical ventilation n = 116	*p*-value
Age, years	53.79 ± 12.90	53.43 ± 12.43	54.39 ± 13.65	0.403
Male, n (%)	323 (60.94)	207 (62.92)	116 (57.47)	0.233
Diabetes, n (%)	149 (28.11)	83 (25.23)	66 (32.84)	0.059
Hypertension, n (%)	166 (31.32)	103 (31.31)	63 (31.34)	0.993
Obesity, n (%)	298 (75.25)	192 (73.85)	106 (77.94)	0.370
Ischemic cardiopathy, n (%)	37 (6.98)	21 (10.45)	16 (4.86)	0.014
Pulmonary disease, n (%)	84 (15.85)	52 (15.81)	32 (15.92)	0.972
Thyroid disease, n (%)	22 (4.15)	10 (3.04)	12 (5.97)	0.101
Hepatopathy, n (%)	19 (3.58)	14 (4.26)	5 (2.49)	0.288
HIV, n (%)	3 (0.57)	2 (0.61)	1 (0.5)	0.869
Asthma (%)	12 (2.26)	7 (2.13)	5 (2.49)	0.787
COPD, n (%)	9 (1.7)	4 (1.22)	5 (2.49)	0.272
*Hospitalary parameters*				
Length of hospital stay, d	17 [10–29]	25 [17–36]	9 [7–13]	< 0.001
Duration of IMV	13 [9–20]	13 [9–20]	–	–
Arterial oxygen saturation, %	85 [73–90.6]	81.5 [69.1–90]	86.7 [81–91]	< 0.001
PaO_2_%	52.75 [40.35–66.1]	49.7 [36.1–65.3]	55 [45.8–67.5]	0.010
PaO_2_/FiO_2_ ratio	156.35 [105.04–212.85]	129.52 [90.93–176.09]	226.42 [172.99–264.76]	< 0.001
ARDS mild, n (%)	72 (26.18)	28 (14)	44 (58.67)	< 0.001
ARDS moderate-severe, n (%)	203 (73.82)	172 (86)	31 (41.33)	
*Body composition at 3 months*				
Weight, kg	77 [68–86.8]	76.1 [67–85]	79 [69–87.3]	0.112
Height, cm^2^	161.69 ± 8.88	161.37 ± 8.67	162.23 ± 9.2	0.279
Body mass index, kg/m^2^	29.05 [25.9–32.86]	29.05 [25.63–32.65]	29.031 [26.34–33.2]	0.431
Fat Mass, %	24.13 [18.42–31.31]	24.13 [17.87–31.62]	24.13 [19.68–31.03]	0.553
FFMI, kg	19.92 ± 3.21	19.93 ± 3.10	19.89 ± 3.43	0.917
ASMM, kg	20.33 [17.31–23.19]	20.12 [17.37–22.89]	20.86 [17.24–23.77]	0.091
ASMMI, kg	7.71 [7.01–8.51]	7.68 [6.99–8.42]	7.84 [7.10–8.64]	0.129
Phase angle, °	6.1 [5.3–6.9]	5.9 [5–6.7]	6.55 [5.7–7.2]	< 0.001
Impedance index, 200/50 kHz	0.8 [0.77–0.83]	0.81 [0.78–0.83]	0.78 [0.76–0.81]	< 0.001
Handgrip strength, kg	23.6 ± 9.32	22.07 ± 9.11	26.09 ± 9.15	< 0.001
MIP, cmH_2_O	94.07 ± 26.89	92.64 ± 25.71	96.38 ± 28.61	0.137
MIP < 80% pred, n (%)	117( 22.08)	74( 22.49)	43 ( 21.39)	0.767
MEP, cmH_2_O	115.19 ± 35.46	114.25 ± 33.96	116.7 ± 37.82	0.460
MEP < 80% pred, n (%)	404(76.23)	255 (77.51)	149 (74.13)	0.375
Low muscle mass, n (%)	130 (24.53)	92 (27.96)	38 (18.91)	0.019
Low muscle strength, n (%)	251 (47.36)	192 (58.36)	59 (29.35)	< 0.001
Sarcopenia, n (%)	98 (18.49)	74 (22.49)	24 (11.94)	0.069

HIV, Human immunodeficiency virus; COPD, Chronic Obstructive Pulmonary Disease; VMI, Ventilation mechanical invasive; Pa0_2_, Arterial partial pressure of oxygen; PaO2/FiO_2_, Arterial partial pressure of oxygen/ Fraction of inspired oxygen; ARDS,Acute Respiratory Distress Syndrome; FFMI, Fat Free Mass Index; ASMM, Appendicular skeletal muscle mass, ASMMI, Appendicular skeletal muscle mass index, MIP, Maximum inspiratory pressures; MEP, Maximum expiratory pressures

The risk factors associated with sarcopenia were > 60 years of age, diabetes, obesity, IMV, duration of IMV, and hospital stay > 7 days (Table 2). The multivariate model showed that age > 60 years, obesity and the interaction between prolonged hospital stay and IMV were associated with a higher risk of sarcopenia (Table 3).

**Table 2:** Risk factors associated to sarcopenia in Post-COVID patients. Bivariate model

	OR	CI 95%	*p*-value
> 60 years old	3.98	2.46–6.45	< 0.001
Male	0.96	0.61–1.50	0.868
Diabetes	1.63	1.03–2.60	0.037
Hypertension	1.27	0.80–2.02	0.300
Obesity	2.17	1.06–4.43	0.033
Ischemic cardiopathy	1.03	0.43–2.42	0.945
Pulmonary disease	1.14	0.63–2.05	0.653
Thyroid disease	0.68	0.19–2.36	0.551
Hepatopathy	1.18	0.38–3.64	0.770
VIH	8.97	0.80–100.03	0.074
Asthma	0.39	0.05–3.09	0.376
COPD	3.63	0.95–13.79	0.058
*Hospitalary parameters*			
IMV	2.14	1.29–3.52	0.003
Duration of IMV, d	1.05	1.01–1.09	0.003
Prolonged length of hospital stay, > 7 d	3.04	1.64–5.63	< 0.001
Arterial oxygen saturation, %	1.00	0.98–1.02	0.797
PaO_2_, %	0.99	0.97–1.00	0.415
PaO_2_/FiO_2_ ratio	0.99	0.99–1.00	0.054
ARDS mild	1	Reference	
ARDS moderade-severe	1.71	0.78–3.74	0.174

HIV, Human immunodeficiency virus; COPD, Chronic Obstructive Pulmonary Disease; IMV, Invasive Mechanical Ventilation; Pa0_2_, Arterial partial pressure of oxygen; PaO2/FiO_2_, Arterial partial pressure of oxygen/ Fraction of inspired oxygen; ARDS, Acute Respiratory Distress Syndrome

**Table 3:** Risk factors associated to sarcopenia in Post-COVID patients. Multivariate model

	OR	CI 95%	*p*-value
Age > 60 years	4.91	2.26–10.63	< 0.001
Obesity	3.73	1.21–11.54	0.022
IMV* Prolonged length of hospital stay, > 7 d	2.92	1.21–7.02	0.016

IMV, Invasive mechanical invasive *Adjusted for sex and, diabetes, VIH, COPD and, PaO_2_/FiO_2_ ratio

## Discussion

There were two main findings from this study. The first involved the differences in body composition alterations, especially skeletal muscle abnormalities, in post-COVID-19 survivors who had undergone IMV and those who had not. The second was the identification of risk factors associated with sarcopenia.

In our population, 47.36% had low muscle strength. This was an independent predictor of poor patient outcomes such as persistent fatigue, diminished capacity to perform activities of daily living, pulmonary function, physical functioning, prolonged hospital stay, poor health-related quality of life, and mortality in other populations [11, 12, 15, 16, 30–32]. Cheval et al. also showed that muscle strength was an independent risk factor for COVID-19 hospitalization (OR: 0.64, 95% CI: 0.45–0.92, *p* = 0.015) [33].

In addition, at six months after acute COVID-19, the most frequent consequences were fatigue or muscle weakness [6]. In our population, 24.5% had low muscle mass; this was another important independent predictor of worsening capacity to perform activities of daily living, falls, and mortality [16, 34].

Sarcopenia is defined as the presence of both low muscle mass and low muscle function and increases the risk of adverse outcomes such as physical disability and mortality [14, 35, 36]. In our population, 18.49% had sarcopenia. One of the factors that may contribute to the development of sarcopenia in post-COVID-19 patients is age. It has been reported that individuals more than fifty years old undergo a 1–2% loss of muscle mass and a loss of muscular strength of about 1.5–5% per year [37]. In adults > 60 years of age, the prevalence of sarcopenia was 10% [38]. In our study, the risk of sarcopenia in subjects > 60 years old increased five times (OR: 4.91, 95% CI: 2.26–10.63, *p* < 0.001) adjusted to confounding variables.

Another important risk factor for sarcopenia is prolonged hospitalization. Patients with moderate to severe COVID-19 had prolonged hospital stays. Those requiring supplemental oxygen remained a median of 14 days (10–18), while those with high-flow nasal cannula for oxygen therapy or IMV were in the hospital for a median of 35 days (22–51) [6]. In our study, the mean length of hospital stay was 25 days (17–36) for IMV subjects and nine days (7–13) for those without IMV. Patients with prolonged hospital stays (> 7 days) were at 3.04 times greater risk of developing sarcopenia (OR: 3.04; 95%CI: 1.64–5.63, *p* < 0.001) in the bivariate model. Alley et al. showed that both men and women with eight or more days of hospitalization had declines of lean mass (− 0.81 kg and − 0.43 kg, respectively) and loss of knee extensor strength (− 7.89 Nm and − 7.47 Nm, respectively) [18].

Another significant risk factor for sarcopenia and skeletal muscle disorders is IMV. In patients who require IMV, infection, prolonged hyperglycemia, malnutrition, use of neuromuscular blocking agents, and use of corticosteroids may also contribute to low muscle strength, in both skeletal and respiratory muscles [13, 39–41]. In our population, the mean duration of IMV was 13 [9–20] days, and every day increase 5% the risk of sarcopenia in a bivariate model (OR: 1.05, 95% CI: 1.01–1.09, *p* = 0.003). Besides, subjects who underwent IMV during hospitalization had lower phase angle and higher impedance index. Increased values for these variables are considered to be indicators of higher cellularity, membrane integrity, cellular function, nutritional status, hydric status, and prognosis in other populations [42–45].

We also observed that IMV patients had lower muscle mass and lower muscle strength than patients not exposed to IMV. Several studies have shown that both low muscle strength (OR: 1.51, 95% CI: 1.34–1.70), and low muscle mass (OR: 3.19, 95% CI: 1.29–7.92) are associated with a diminished capacity to perform normal activities and a worse prognosis [11, 16, 34]. Moreover, IMV subjects were at greater risk of developing sarcopenia than subjects not managed with IMV in the bivariate model. Huang et al., showed that at 6 months after acute infection of COVID-19 subjects who requiring high-flow nasal cannula, non-invasive mechanical ventilation, or IMV during their hospital stay had more risk to fatigue or muscle weakness (OR: 2.69, 95% CI: 1.46–4.96) and, lower distance walked (β: − 32.50, 95% CI: − 51.40 to − 13.60) than subjects who not requiring supplemental oxygen.

It is estimated that between 60 and 80% of IMV patients had diaphragmatic weakness [19, 40], which is associated with prolonged IMV and mortality [39, 46]. Global respiratory muscle strength is evaluated by maximum inspiratory pressure (MIP), which estimates the strength of the inspiratory muscles (diaphragm), and maximal expiratory pressure MEP, which estimates the strength of the intercostal and abdominal muscles. In COVID-19 patients 30 days after being discharged from the hospital, Huang et al. demonstrated low respiratory muscle strength in both inspiratory and expiratory muscles (52.9% had MIP < 80% of predicted and 23.5% had MEP < 80% of predicted, respectively) [47]. In our population, three months after acute COVID-19 infection we found that 22.08% had low strength of the inspiratory muscles, and 76.23% had low strength of the expiratory muscles. There was no difference in declining respiratory muscle strength between those managed with IMV and those who were not. This finding concurred with Huang et al. report [47]: pulmonary function is reduced in COVID-19 survivors. Salem et al. showed decreases in total lung capacity, forced vital capacity (FVC), forced expiratory volume (FEV_1_), FEV_1_/FEV, and diffusing capacity for carbon monoxide [48]. Prior evidence in other populations as well as in COVID-19 patients has shown that pulmonary rehabilitation improves respiratory function and functional capacity [49, 50].

Another of the risk factors associated with sarcopenia involves the pro-inflammatory elements [51, 52]. In acute COVID-19, elevated levels of pro-inflammatory markers have been documented, and they are higher in patients with severe COVID-19 and non-survivors than in stable subjects [2, 3]. Elevation of these pro-inflammatory cytokines like IL-1β, IL-6, TNF-α, interferon-γ, as well CRP and NF-κB, induce muscle fiber proteolysis and decrease protein synthesis [17, 20, 53, 54]. In addition, pro-inflammatory factors are associated with lower skeletal muscle strength and low muscle mass and increased risk of sarcopenia [51, 52]. Pre-existing comorbidities also increase the risk of sarcopenia; in our study, after adjusting for confounding variables, obesity was associated with greater risk of sarcopenia, similar to results found previously [21, 55]. Obesity has also been associated with increased inflammatory markers, production of reactive oxygen species, insulin resistance, and fat infiltration, promoting muscle catabolism and inhibiting muscle anabolic pathways [56].

In the case of diabetes, this was associated to sarcopenia in bivarite model but not in multivarite, however the evidence in other studies showed that insulin resistance increases protein degradation and reduces protein synthesis. This initiates a vicious cycle since loss of muscle mass has a negative impact on insulin resistance, and muscle is a target tissue of insulin [57].

Moreover, malnutrition plays an essential role in the loss of muscle mass during hospitalization. At the same time, the most common digestive symptoms in patients with COVID-19 are nausea, vomiting, diarrhea, and loss of appetite [58]. These symptoms may impact nutritional status and body composition with loss of muscle mass. In COVID-19 hospitalized patients, malnutrition was 42.1%, which increased to 66.7% in patients admitted to the intensive care unit [59]. A meta-analysis performance by Ojo et al. in critically ill patients with COVID-19 showed that early enteral nutrition reduced 11% the risk of mortality (RR: 89, 95%CI; 0.79 to 0.99) and a reduction in SOFA score [60].

### Strength and limitations of the study

This study has the inherent limitations of a cross-sectional study: It is impossible to assess causality, and we cannot know the incidence of sarcopenia during hospitalization for COVID-19. Another limitation is that we did not evaluate inflammation markers to determine their association with sarcopenia in post-COVID-19 subjects. However, among its strengths, this is the first study in post-COVID-19 patients that assesses body composition comparing patients managed with and without IMV in association with risk factors for sarcopenia using a multivariate prediction model adjusted for confounding variables. This provides us with a profile of the musculoskeletal alterations of these subjects after their hospitalization.

## Conclusion

In post-COVID-19 patients, the interaction between prolonged length of hospital stay > 7 days and IMV and obesity lead to a greater risk of sarcopenia at three months after severe or moderate COVID-19 infection.

## Data Availability

The datasets generated and/or analysed during the current study are not publicly available due to the fact that individual privacy could be compromised but are available from the corresponding author on reasonable request.

## Abbreviations

ASMM: Appendicular skeletal muscle mass
CI: Confidence intervals
COVID-19: Coronavirus infection disease 2019
IMV: Invasive mechanical ventilation
MIP: Maximum inspiratory pressure
MEP: Maximum expiratory pressure
OR: Odds ratio
PaO_2_/FiO_2_: Arterial partial pressure of oxygen/Fraction of inspired oxygen
SARS-CoV-2: Severe acute respiratory syndrome-coronavirus-2
